# Predictive Factors in Development of Postoperative Delirium in Chronic Subdural Hematomas: A Prospective Multicenter Study

**DOI:** 10.3390/jcm15093412

**Published:** 2026-04-29

**Authors:** Ismail Zaed, Salvatore Chibbaro, Francesco Marchi, Luca Ricciardi, Leonardo Di Cosmo, Charles Henry Mallereau, Guillaume Dannhoff, Julien Todeschi, Mario Ganau, Davide Milani, Andrea Cardia

**Affiliations:** 1 Department of Neurosurgery, Neurocenter of Southern Switzerland, Ente Ospedaliero Cantonale, 6900 Lugano, Switzerland; 2 Department of NESMOS, Sapienza University, 00185 Rome, Italy; 3 Neurosurgery Unit, Department of Medical and Surgical Sciences and Neurosciences, Siena University Hospital, Azienda Ospedaliera-Universitaria, 53100 Siena, Italy; 4 Department of Neurosurgery, Hautepierre Hospital, Strasbourg University Hospital, 67000 Strasbourg, France; 5 UOC di Neurochirurgia, Azienda Ospedaliera Universitaria Sant’Andrea, Dipartimento NESMOS, Sapienza Università di Roma, 00185 Rome, Italy; 6 School of Medicine, Humanitas University, Via Rita Levi Montalcini 4, Pieve Emanuele, 20072 Milan, Italy; 7 Nuffield Department of Clinical Neurosciences, University of Oxford, Oxford OX3 9DU, UK

**Keywords:** subdural hematoma, postoperative delirium, postoperative complications, risk factors

## Abstract

**Introduction**: Chronic subdural hematoma (CSDH) is a complex disease with an overall incidence of 1.7–20.6 per 100,000 persons per year and is more commonly encountered in the elderly population. It is projected to be one of the most common neurosurgical procedures. Postoperative delirium is a common complication associated with the elderly, causing increased morbidity and prolonged hospital stay. However, its risk factors in chronic subdural hematoma patients have not been well studied. **Methods**: A total of 202 consecutive patients with chronic subdural hematoma at different neurosurgical centers in Europe between January 2023 and June 2025 were enrolled. Various clinical indicators were analyzed to identify independent risk factors for postoperative delirium using univariate and multivariate regression analyses. **Results**: Out of the 202 patients (age, 71 (IQR, 18); female-to-male ratio, 1:2.7) studied, 63 (31.2%) experienced postoperative delirium. Univariate analysis identified age (*p* < 0.001), gender (*p* = 0.014), restraint belt use (*p* < 0.001), electrolyte imbalance (*p* < 0.001), visual analog scale (VAS) pain score (*p* < 0.001), hematoma thickness (*p* < 0.001), midline shift (*p* < 0.001), hematoma side (*p* = 0.013), hematoma location (*p* = 0.018), and urinal catheterization (*p* = 0.028) as significant factors. Multivariate regression analysis confirmed the significance of restraint belt use (B = 7.657, *p* < 0.001), electrolyte imbalance (B = −3.993, *p* = 0.001), VAS pain score (B = 2.331, *p* = 0.016), and midline shift (B = 0.335, *p* = 0.007). Hematoma thickness and age had no significant impact. **Conclusions**: Increased midline shift and VAS pain scores, alongside restraint belt use and electrolyte imbalance, elevate delirium risk in chronic subdural hematoma surgery. Our prediction models may offer a reference value in this context.

## 1. Introduction

Chronic subdural hematoma (cSDH) is a common neurosurgical condition, particularly affecting the elderly population, with an increasing incidence in aging societies [1,2]. Characterized by the accumulation of blood in the subdural space, cSDH often presents with nonspecific symptoms such as cognitive decline, gait disturbances, and focal neurological deficits [3]. Surgical evacuation remains the cornerstone of treatment, typically yielding favorable neurological outcomes. However, a significant proportion of patients experience postoperative complications that can adversely affect recovery, among which postoperative delirium (POD) is notably prevalent and clinically impactful [3].

Postoperative delirium is an acute neuropsychiatric syndrome characterized by fluctuating disturbances in attention, awareness, and cognition [4]. In neurosurgical patients, especially those undergoing procedures for cSDH, POD is associated with longer hospital stays, increased morbidity, reduced functional recovery, and higher rates of institutionalization. Despite its frequency and clinical relevance, the pathophysiology of POD remains incompletely understood, and its prediction in the postoperative setting continues to challenge clinicians [5,6].

Several factors have been proposed as contributors to the development of POD in cSDH patients, including advanced age, pre-existing cognitive impairment, use of anticoagulant or antiplatelet medications, perioperative infection, and the type of anesthesia administered [4,5,6]. However, evidence remains heterogeneous and often retrospective in nature, limiting the generalizability of findings and the implementation of effective preventative strategies.

This prospective multicenter study aims to identify and analyze predictive factors associated with the development of postoperative delirium in patients undergoing surgical treatment for chronic subdural hematomas. By leveraging multicenter data across diverse populations and standardized diagnostic criteria, we seek to improve understanding of POD in this context and to inform future risk stratification and perioperative management strategies.

## 2. Material and Method

### 2.1. Study Design and Setting

This was a prospective, multicenter observational study conducted across several neurosurgical centers in Europe between different countries, such as Switzerland, Italy, the United Kingdom, and France. The data were collected for a period of 18 months from January 2023 to June 2025. The aim was to evaluate clinical, demographic, and perioperative factors associated with the development of postoperative delirium in patients undergoing surgical treatment for chronic subdural hematomas (cSDH).

### 2.2. Patient Population

Adult patients (aged ≥ 18 years) diagnosed with cSDH and scheduled for surgical evacuation were consecutively enrolled. Diagnosis of cSDH was based on clinical presentation and neuroimaging findings, obtained by a non-contrast CT. Exclusion criteria included acute subdural hematoma, previous craniotomy for cSDH within the past 6 months, known psychiatric illness unrelated to delirium (e.g., schizophrenia), terminal illness with life expectancy < 30 days, or refusal to consent.

### 2.3. Data Collection

Data were collected prospectively using standardized case report forms from a surgeon responsible for each center and included the following:

Demographic and baseline clinical variables included age, sex, alcoholism history, hepatic insufficiency, hypertension, diabetes, known dementia, antiplatelet/anticoagulant exposure, and baseline cognitive vulnerability when available from the clinical chart.

Binary predictors were coded as 1 = present and 0 = absent. Electrolyte imbalance was defined as at least one clinically relevant perioperative abnormality within 24 h before surgery or during the first postoperative day (serum sodium < 135 or >145 mmol/L, potassium < 3.5 or >5.1 mmol/L, or corrected calcium below the local laboratory lower limit). Hepatic insufficiency was defined by a documented chronic liver disease with laboratory or imaging evidence of impaired synthetic or excretory function. Restraint belt use was coded as present when a physical restraint was applied at any time during the first 48 postoperative hours. Urinary catheterization was coded as present when an indwelling urinary catheter was maintained beyond the immediate post-anesthesia recovery period.

Continuous predictors were entered in their native scale and were not dichotomized. Specifically, VAS pain score was analyzed per 1-point increase and midline shift per 1 mm increase. Age and hematoma thickness were also retained as continuous variables during the multivariable modeling stage.

Radiological variables included hematoma side, maximum thickness, midline shift, frontal/parietal extension, and bilaterality. Surgical variables included burr-hole craniostomy versus craniotomy, general versus local anesthesia, and postoperative drain placement.

### 2.4. Outcome Measure

The primary outcome was the development of postoperative delirium, assessed within the first 7 days following surgery. Delirium was diagnosed using the Confusion Assessment Method (CAM) or the CAM-ICU for intubated patients, administered by trained personnel. Assessments were conducted at least twice daily.

### 2.5. Statistical Analysis

Categorical variables were compared using the chi-square test or Fisher’s exact test, as appropriate, and continuous variables using the Student *t*-test or Mann–Whitney U test according to distribution. Variables associated with POD at *p* < 0.10 in univariate analyses, together with clinically relevant covariates collected a priori (known dementia, anesthesia type, and surgical approach), were considered for multivariable logistic regression.

To reduce overfitting, the final model was built using a backward step-down procedure starting from the candidate set while retaining clinically essential covariates. Binary predictors were entered with the reference category coded as 0 (absence of the feature). Multicollinearity was assessed through the variance inflation factor (VIF), with VIF > 5 considered potentially problematic. Model calibration was explored with the Hosmer-Lemeshow goodness-of-fit test and calibration plot, whereas discrimination was summarized with the area under the receiver operating characteristic curve (AUC). For illustrative bedside performance, a prespecified probability threshold of 0.30 was used to derive sensitivity, specificity, accuracy, precision, and F1 score.

Analyses were performed with R version 4.1.1 (R Foundation for Statistical Computing, Vienna, Austria). A two-sided *p*-value < 0.05 was considered statistically significant.

### 2.6. Ethical Consideration

This study was conducted according to the Ethical Principles for Medical Research Involving Human Subjects stated in the Declaration of Helsinki issued in 2004 and its further revisions made in 2008 and 2013. To report our results, we followed the recommendations of the STROCCS (Strengthening the reporting of observational cohort studies in surgery) statement [6,7,8]. The study was approved by the IRB of the French College of Neurosurgery (reference number: IRB00011687, approval date: 5 December 2022).

## 3. Results

### 3.1. Patient Characteristics

A total of 202 patients undergoing surgical treatment for cSDH were prospectively included. Median age was 71 years (interquartile range, 18 years), and 146 patients (72.3%) were male. POD developed in 63 patients (31.2%).

Compared with the non-POD group, patients who developed POD were older and more frequently had a history of alcoholism, hepatic insufficiency, electrolyte imbalance, restraint belt use, and urinary catheterization. Mean postoperative VAS pain score was also higher in the POD group. Pre-existing dementia, general anesthesia, and surgical approach were recorded in the cohort; these variables showed a numerical imbalance toward the POD group but did not remain independently associated with POD after adjustment. Baseline demographics and clinical characteristics are summarized in Table 1. Patients who developed postoperative delirium were significantly older (*p* < 0.001). Gender distribution also differed significantly between groups (*p* = 0.014), with males showing a small, yet statistically significant, higher incidence of POD.

Several clinical variables have been documented to define a correlation with POD. It has been found that delirium is associated with several factors, among which are electrolyte imbalance, use of restraint belts, and urinary catheterization. Patients requiring physical restraint demonstrated a markedly higher risk of developing POD (*p* < 0.001). Similarly, electrolyte disturbances were significantly more frequent among delirious patients (*p* < 0.001). Urinary catheterization was also associated with POD development (*p* = 0.028).

Pain severity measured using the visual analog scale (VAS) pain score was significantly higher in patients who developed delirium (*p* < 0.001).

### 3.2. Radiological and Surgical Characteristics

Radiological findings and surgical characteristics are presented in Table 2. Hematoma-related parameters showed several associations with delirium development.

In univariate analysis, greater hematoma thickness was significantly associated with POD (*p* < 0.001). Midline shift was also strongly correlated with delirium occurrence (*p* < 0.001). Additionally, hematoma laterality (*p* = 0.013) and hematoma location (*p* = 0.018) demonstrated significant relationships with postoperative delirium.

These findings suggest that structural brain displacement and hematoma characteristics may contribute to postoperative cognitive vulnerability.

### 3.3. Multivariate Analysis

In the multivariable model, restraint belt use, electrolyte imbalance, VAS pain score, and midline shift remained independently associated with POD. All the data have been summarized in Table 3. Importantly, after verification of coding and reference categories, electrolyte imbalance showed a positive coefficient, consistent with both the univariate analysis and the raw event rates.

No evidence of severe multicollinearity was found (all VIF values < 2.2), and no coefficient instability suggestive of relevant data separation was observed after refitting the model with Firth-penalized sensitivity analysis, which yielded the same direction and significance of the main predictors. Model calibration was acceptable (Hosmer-Lemeshow *p* = 0.47), and discrimination was good (AUC 0.86, 95% CI 0.80–0.91).

At the prespecified probability threshold of 0.30, the model yielded 50 true positives, 13 false negatives, 114 true negatives, and 25 false positives, corresponding to an accuracy of 81.2%, sensitivity of 79.4%, specificity of 82.0%, precision of 66.7%, and F1 score of 72.5%.

## 4. Discussion

Postoperative delirium (POD) represents a frequent and clinically significant complication following neurosurgical procedures, particularly in elderly populations [7,8]. Chronic subdural hematoma (cSDH) predominantly affects older patients with multiple comorbidities, making this population especially vulnerable to perioperative cognitive disturbances [2,7]. In this prospective European cohort of 202 patients undergoing surgical evacuation of cSDH, postoperative delirium occurred in 31.2% of patients, highlighting the substantial clinical burden associated with this complication. Our findings confirm that POD, as in the other case series reported [5,6], is multifactorial and influenced by a combination of patient-related, perioperative, and radiological factors.

One of the most relevant observations of our study is the identification of restraint belt use as the strongest independent predictor of postoperative delirium. This result could be seen as biased. Physical restraints are frequently applied in clinical practice to prevent falls or protect surgical drains and catheters. This could increase not only the agitation but also the confusion of the patients. However, previous studies have demonstrated that physical restraints may exacerbate psychological distress and contribute to sensory deprivation and disorientation [9,10]. Our results support this concern, suggesting that restraint use may either directly contribute to delirium development or represent a marker of early behavioral disturbance preceding overt delirium. These findings emphasize the importance of minimizing restraint use whenever possible and implementing alternative strategies such as enhanced monitoring, early mobilization, and non-pharmacological delirium prevention protocols [11].

Another important finding is the significant association between electrolyte imbalance and postoperative delirium. Electrolyte disturbances, particularly hyponatremia and other metabolic abnormalities, are known to impair neuronal function and disrupt cerebral homeostasis [6]. Patients with cSDH are often elderly and frequently present with dehydration, polypharmacy, or chronic comorbidities that predispose them to metabolic instability. Our findings reinforce the need for careful perioperative metabolic monitoring and early correction of electrolyte disturbances as part of delirium prevention strategies.

The role of postoperative pain, as reflected by higher VAS pain scores, also emerged as an independent predictor of delirium [12,13]. Higher VAS pain scores independently predicted POD, reinforcing the concept that untreated postoperative pain is not benign. Pain contributes to sympathetic activation, sleep fragmentation, and inflammatory stress, all of which can precipitate delirium. This observation supports structured analgesic pathways with regular pain assessment while avoiding oversedation.

Pain can contribute to sleep disturbance, sympathetic activation, and neuroinflammatory responses, all of which may increase susceptibility to cognitive dysfunction. In addition, inadequate pain control may lead to agitation, which can further complicate postoperative management. These findings highlight the importance of optimized analgesic protocols in patients undergoing cSDH surgery, balancing effective pain control while avoiding excessive sedative medications that may themselves precipitate delirium.

From a radiological perspective, midline shift remained an independent predictor of delirium after adjustment for confounding variables. Midline shift reflects the degree of mass effect exerted by the hematoma on surrounding brain structures and may indicate more pronounced cortical and subcortical dysfunction. Greater mechanical displacement of brain tissue may disrupt neural networks involved in attention, arousal, and cognition, thereby predisposing patients to postoperative cognitive disturbances. The association between midline shift and POD deserves mechanistic consideration. Midline shift is not merely a radiological severity marker; it likely reflects distortion of cortical-subcortical networks involved in arousal, attention, and executive integration. Mechanical stress may also interact with blood–brain barrier dysfunction, local cytokine release, and microglial activation, thereby facilitating a neuroinflammatory milieu that lowers the threshold for delirium in susceptible older patients. Interestingly, although hematoma thickness was significantly associated with delirium in the univariate analysis, it did not remain an independent predictor in the multivariate model, suggesting that the degree of brain displacement may be a more relevant parameter than hematoma size alone [14].

Age is widely recognized as a major risk factor for delirium in many surgical populations. In our cohort, advanced age was significantly associated with delirium in univariate analysis but did not retain significance in the multivariate model [15]. This finding may indicate that age-related vulnerability is mediated through other clinical factors such as metabolic disturbances, comorbidities, or increased sensitivity to perioperative stressors.

Taken together, these findings highlight the multifactorial pathophysiology of postoperative delirium, involving structural brain factors, metabolic disturbances, and perioperative management variables. Importantly, several of the identified predictors—such as electrolyte imbalance, pain management, and restraint use—represent potentially modifiable factors, suggesting opportunities for targeted preventive strategies.

### 4.1. Clinical Implications

The identification of risk factors for postoperative delirium has important clinical implications for the perioperative management of patients undergoing surgery for chronic subdural hematoma. Early recognition of high-risk patients may allow clinicians to implement preventive interventions such as improved metabolic monitoring, optimized analgesia, and strategies aimed at minimizing physical restraint use. In addition, radiological indicators such as midline shift may help identify patients requiring closer postoperative neurological surveillance.

Multidisciplinary delirium prevention programs, which include early mobilization, sleep optimization, cognitive stimulation, and careful medication management, have shown promising results in other surgical populations. Our findings suggest that similar strategies could be beneficial in neurosurgical patients with cSDH.

Furthermore, predictive models incorporating clinical, radiological, and perioperative factors may help guide risk stratification and individualized postoperative care, potentially reducing morbidity and healthcare resource utilization associated with delirium.

### 4.2. Study Limitations

Several limitations should be acknowledged when interpreting the results of this study. First, although the study was conducted prospectively, it remains an observational study, and therefore, causal relationships between the identified predictors and delirium cannot be definitively established.

Second, variations in clinical practice among participating centers, including postoperative management protocols and delirium assessment routines, may have introduced heterogeneity into the dataset. Although standardized diagnostic criteria were used, interobserver variability in delirium recognition cannot be completely excluded.

Third, certain potential contributors to delirium—such as preexisting cognitive impairment, medication exposure, sleep disturbances, or frailty status—were not comprehensively evaluated and may represent unmeasured confounders.

Finally, although the sample size of 202 patients provides meaningful insights, larger multicenter studies may be required to validate the predictive model and confirm the generalizability of our findings across different healthcare systems.

### 4.3. Future Directions

Future research should focus on prospective validation of delirium prediction models in larger and more diverse neurosurgical populations. Incorporating additional clinical parameters such as frailty indices, baseline cognitive status, and perioperative medication exposure may improve the accuracy of risk stratification tools.

Another promising direction involves the development of multimodal delirium prevention protocols specifically tailored to neurosurgical patients. Such programs may include structured pain management strategies, optimized electrolyte monitoring, early mobilization, and environmental interventions aimed at maintaining circadian rhythm and cognitive orientation.

In addition, advances in neuroimaging and neurophysiological monitoring may provide further insights into the mechanisms linking structural brain displacement and cognitive dysfunction. Understanding the neurobiological pathways underlying delirium could help identify novel therapeutic targets and preventive strategies.

Finally, given the increasing incidence of chronic subdural hematoma in aging populations worldwide, future studies should also evaluate the long-term cognitive and functional outcomes of patients who experience postoperative delirium following cSDH surgery. Such data may help clarify the broader impact of delirium on recovery and quality of life.

## 5. Conclusions

Postoperative delirium is a frequent complication following surgical treatment of chronic subdural hematoma, affecting nearly one-third of patients in this prospective European cohort. Our findings demonstrate that increased midline shift, higher postoperative pain levels, electrolyte imbalance, and the use of restraint belts are independent predictors of delirium development.

Importantly, some traditionally implicated factors, such as age and hematoma thickness, did not independently influence delirium risk after adjustment for confounders, suggesting that modifiable perioperative factors may play a more critical role than previously assumed. These results emphasize the need for careful perioperative management, particularly with respect to pain control, electrolyte monitoring, and the judicious use of physical restraints.

The predictive models derived from this study may serve as a useful reference for identifying high-risk patients and guiding targeted preventive strategies aimed at reducing postoperative delirium and improving overall outcomes in chronic subdural hematoma surgery.

## Figures and Tables

**Table 1:** Baseline Demographics and Clinical Characteristics.

Variable	POD (n = 63)	No POD (n = 139)	Total	*p*-Value
Age (years), mean ± SD	76 ± 8	69 ± 10	71 ± 9	<0.001
Male sex, n (%)	50 (79.4)	96 (69.1)	146 (72.3)	0.014
Alcoholism history, n (%)	18 (28.6)	19 (13.7)	37 (18.3)	0.012
Hepatic insufficiency, n (%)	7 (11.1)	6 (4.3)	13 (6.4)	0.047
Known dementia, n (%)	9 (14.3)	10 (7.2)	19 (9.4)	0.089
Electrolyte imbalance, n (%)	28 (44.4)	20 (14.4)	48 (23.8)	<0.001
Restraint belt use, n (%)	22 (34.9)	5 (3.6)	27 (13.4)	<0.001
Urinary catheterization, n (%)	40 (63.5)	68 (48.9)	108 (53.5)	0.028
VAS pain score, mean ± SD	6.2 ± 1.5	3.8 ± 1.7	4.6 ± 1.9	<0.001
General anesthesia, n (%)	36 (57.1)	67 (48.2)	103 (51.0)	0.241

**Table 2:** Radiological and surgical characteristics.

Variable	POD (n = 63)	No POD (n = 139)	*p*-Value
Hematoma thickness (mm), mean ± SD	19.4 ± 5.1	15.2 ± 4.8	<0.001
Midline shift (mm), mean ± SD	7.1 ± 3.0	4.2 ± 2.6	<0.001
Bilateral hematoma, n (%)	26 (41.3)	33 (23.7)	0.013
Frontal/parietal extension, n (%)	48 (76.2)	86 (61.9)	0.018
Drain placement, n (%)	57 (90.5)	123 (88.5)	0.670

**Table 3:** Multivariate Logistic Regression analysis.

Predictor	B	SE	Adjusted OR	95% CI	*p*-Value
Restraint belt use (1 vs. 0)	2.61	0.64	13.6	3.8–48.5	<0.001
Electrolyte imbalance (1 vs. 0)	1.94	0.56	7.0	2.3–21.0	<0.001
VAS pain score (per 1-point increase)	0.39	0.15	1.48	1.11–1.98	0.008
Midline shift (per 1 mm increase)	0.19	0.07	1.21	1.06–1.38	0.004
Age (per 1-year increase)	0.03	0.02	1.03	0.99–1.08	0.181
Known dementia (1 vs. 0)	0.57	0.42	1.77	0.78–4.00	0.169
General anesthesia (1 vs. 0)	0.18	0.31	1.20	0.66–2.18	0.548
Burr-hole approach (1 vs. 0)	−0.29	0.44	0.75	0.32–1.78	0.521

## Data Availability

The original contributions presented in this study are included in the article. Further inquiries can be directed to the corresponding author.
